# Elevated transmission of upper respiratory illness among new recruits in military barracks in Thailand

**DOI:** 10.1111/irv.12345

**Published:** 2015-10-13

**Authors:** Jens W Levy, Piraya Bhoomiboonchoo, Sriluck Simasathien, Henrik Salje, Angkana Huang, Ram Rangsin, Richard G Jarman, Stefan Fernandez, Chonticha Klungthong, Kittinun Hussem, Robert V Gibbons, In-Kyu Yoon

**Affiliations:** a Department of Virology, Armed Forces Research Institute of Medical SciencesBangkok, Thailand; b Phramongkutklao HospitalBangkok, Thailand; c Department of Epidemiology, Johns Hopkins, Bloomberg School of Public HealthBaltimore, MD, USA

**Keywords:** Human, military, respiratory tract infections, Thailand, transmission

## Abstract

**Background:**

New recruits within military barracks present conditions favorable for the spread of respiratory pathogens. However, respiratory pathogen transmission in such confined settings in the tropics has not been well studied.

**Methods:**

Recruits in four successive Royal Thai Army basic training classes living in military barracks were monitored for the symptoms of influenza-like illness (ILI) or upper respiratory illness (URI). Classes 1 and 2 were also monitored after basic training. Nasal/throat swabs from acute illnesses were collected and tested by influenza RT-PCR (all four classes). In addition, class 1 had multiplex PCR performed along with the analysis of bed locations within the barracks.

**Results:**

Influenza-like illness/upper respiratory illness rates ranged from 4·7 to 6·9 per 100 recruit-weeks in the four classes and generally decreased during the course of basic training (*P* < 0·05 in three of four classes). Rates during basic training were 1·7 (95% CI: 1·29, 2·29) and 2·5 (95% CI: 1·5, 4·1) times higher than after basic training (classes 1 and 2, respectively). In class 1, coronavirus, parainfluenza virus, and rhinovirus were the most commonly identified respiratory pathogens; only one influenza PCR-positive infection was detected in all four classes. Bed locations of URI/ILI cases in class 1 tended to be in closer proximity to each other.

**Conclusion:**

Basic training recruits in military barracks in the tropics had high rates of acute respiratory illnesses with illness patterns consistent with external seeding followed by substantial internal transmission. Our findings may contribute to control measures in similar confined settings both within and outside the military.

## Introduction

Recruitment into the military and congregation of recruits in training camps brings together many people from geographically diverse areas into close living conditions. Similar to other settings, such as college dormitories, sports teams, and cruise ships, the close physical proximity of individuals in military barracks enhances the risk of the transmission of respiratory and enteric pathogens. The congregation of diverse individuals can lead to the introduction of one or more pathogens into a confined population, leading to an outbreak of infections. Institutions that permit frequent, longer term, and intimate contacts among individuals generally can be expected to have higher attack rates.^1^ Other factors such as physical exertion and the prevalence of immunologically naïve individuals can contribute to the unique vulnerability of military recruits.^2^

Militaries are a unique subset of institutions that potentially have higher attack rates than other societal groupings. Military personnel and children may be at greater risk of infection than other groups.^1^ The effect of school closures during epidemics have demonstrated that environments with high people density can function as amplifying arenas for influenza and other respiratory diseases.^3^ The large-scale mobilization of military forces during World War I was a contributing factor to the global influenza pandemic of 1918–1919.^4^,^5^ In the years since then, much research has been done to study respiratory illness among U.S. military recruits. The Commission on Acute Respiratory Diseases conducted studies at United States recruiting camps documenting that rates of respiratory disease among recruits were higher than other ‘seasoned’ military groups.^6^ Miller *et al*. noted a direct correlation between changes in rates of pneumonia and respiratory illness with the number of recruits in training. The number of recruits proved to be an even stronger determinant of infection rates than seasonal factors.^7^ In a more recent study examining influenza vaccine, rates of influenza-like illness (ILI) among recruits were found to be 2–16 times higher than non-recruit service members.^8^ In spite of widespread immunization among U.S. military personnel, influenza outbreaks continue to occur in crowded military settings.^9^,^10^ More recently, human adenovirus (H-AdV) has been a predominant cause of febrile respiratory illness in the U.S. military.^11^

While more is known about the respiratory illness experience of U.S. military personnel, little is known about the experience of military populations of other countries in the tropics and Asia, an area that is very important in the global ecology of influenza.^12^ Vaccination uptake in developing nations is considerably less than it is in developed countries and is not mandatory in the militaries of many countries including Thailand. Furthermore, the prevailing circulating viruses are unknown. Influenza which has well defined seasonality in temperate climates is far more difficult to characterize in the middle latitudes of the tropics.^13^

We report the results of a respiratory illness study among new military recruits at a Royal Thai Army (RTA) barracks in Bangkok, Thailand. These results were complemented by laboratory testing to confirm infection including multiplex PCR.

## Methods

Groups of new RTA conscripts undergo an intensive 10-week basic training during which they are housed together in a single large barracks with one large sleeping quarters. After basic training, the recruits are permitted to go on leave for approximately 10 days. Upon return from leave, some recruits are retained in the camp but live in smaller barracks, while others are assigned elsewhere. In this study, newly enlisted RTA soldiers at least 18 years of age at an RTA training center in Bangkok were eligible to participate. Those with suspected tuberculosis or immunocompromising conditions such as acquired immune deficiency syndrome, lymphoma or leukemia were ineligible. The current study included recruits from four consecutive classes (basic training initiated in May 2013, November 2013, May 2014 and November 2014, respectively) from the same training camp. The study was approved by the Institutional Review Boards of the Royal Thai Army in Bangkok, Thailand, and the Walter Reed Army Institute of Research (WRAIR). Written informed consent was obtained from all subjects.

Individuals giving informed consent were enrolled into the study and subsequently followed for respiratory illness. At enrollment, subjects were instructed to report to the RTA training center medical unit if they had respiratory symptoms. Furthermore, the camp's medical corpsman periodically reminded recruits to report any illnesses. At the time of illness, a history and physical exam was completed by RTA medical staff. Subjects with illnesses that qualified as an upper respiratory illness (URI) or ILI had a throat and nasal swab collected for PCR testing, and a rapid test was carried out to inform clinical care. A URI was defined as an illness with at least two of the following: (i) runny nose or sneezing; (ii) stuffy nose (i.e. congestion); (iii) sore throat, hoarseness or difficulty swallowing; (iv) cough; (v) swollen or tender glands in the neck (i.e. cervical lymphadenopathy); and (vi) fever or abnormal temperature. ILI was defined as an acute onset respiratory illness with a measured temperature (oral) >38°C (100·5°F) with a cough or sore throat. In the first recruit class (designated as class 1), the location of beds belonging to each individual was mapped to evaluate spatio-temporal clustering of illnesses.

### Laboratory

Nasal and throat swabs were tested for influenza viruses using real-time reverse transcriptase polymerase chain reaction (rRT-PCR) using the U.S. CDC protocol.^14^,^15^ In addition, acute samples collected during basic training of class 1 were tested by multiplex real-time PCR using Fast Track Diagnostics (FTD) respiratory pathogens 33 kit (FTD, Esch-sur-Alzette, Luxembourg) according to the manufacturer's instruction to identify a broader spectrum of pathogens present in the class. Resource limitations permitted multiplex PCR testing only on the one class.

### Statistical analysis

Incidence rates for respiratory illness during basic training were calculated using the reported number of URI or ILIs divided by the recruit-weeks observed over the time period between enrollment and the date the recruits were allowed to go on leave. For classes 1 and 2, incidence rates were also calculated for time periods after the end of basic training. Post-basic training was defined as the period beginning with the first full week after recruits returned from leave following basic training (see Appendix S1 for specific time intervals for the class surveillance periods). Confidence intervals for incidence rates and ratios were calculated using formulae from Rothman and Greenland (using the epiR package in r).^16^ All analyses were performed using r version 3.0.2 (R Foundation for Statistical Computing, Vienna, Austria).

The Cochrane–Mantel–Haenszel test was used for linear trend of differences in row means (vcdExtra package in r) to evaluate trend over the weeks between enrollment and the leave after basic training. While rates of infections in sequestered recruits may be expected to increase initially, then decrease as susceptible individuals are depleted, given that enrollment occurred after basic training was initiated (up to 3 weeks for some classes), we used the Mantel-Haenszel chi-square test to evaluate the *P*-for-trend to demonstrate a preponderance of a decrease in the rate over time as evidence that infections came from within the recruit class (i.e. seeded from entry). One-week intervals were used beginning with the week that enrollment occurred, and ending with the week in which recruits went on post-basic training leave. The *P*-for-trend during this last week, however, included some amount of time when the camp was largely unpopulated. For this reason, we included a *P*-for-trend from the enrollment week to the last full week prior to dismissal for post-basic training leave.

To evaluate the transmissibility of infections by close contact (i.e. from droplet transmission), we evaluated whether the bed location, available only for class 1, of those with URI/ILI were clustered using the τ(*d*) method adapted from Salje *et al*.^17^ Using this method, we compared the probability that two individuals had beds within distance *d* meters apart given they had a respiratory illness within a week of each other relative to the probability that any two individuals had beds *d* meters apart. Values of τ(*d*) > 1 indicate spatial clustering of cases at that distance. The estimator we used for τ(*d*) can be found in the Appendix S1. Confidence intervals were calculated using a bootstrap method where all individuals were resampled with replacement and τ(*d*) recalculated over 500 iterations. Ninety-five per cent confidence intervals were calculated from the 2·5 and 97·5 percentiles of the resultant distribution.

## Results

The May 2012 recruit class arrived at the barracks between May 1 and 3, 2012 (class 1); basic training began on May 4. We enrolled 122 of the 131 (93·1%) class 1 recruits into the study on May 14. The recruits were allowed to go on leave on July 17 after basic training. Upon return on July 27, 2012, 58 subjects remained in the camp posted to smaller barracks, while the remainder were assigned elsewhere. The November 2012 recruit class arrived between November 1 and 3, 2012 (class 2); basic training began on November 4. We enrolled 113 of 119 (95·0%) class 2 recruits on November 20. The recruits were allowed to go on leave on January 20, 2013. Sixty-one returned on January 30 and were posted to smaller barracks with the remainder posted elsewhere. The May 2013 class arrived between May 1 and 3, 2013 (class 3). We enrolled 105 (94%) of 116 class 3 recruits on May 31. The recruits were allowed to go on leave beginning July 11. The November 2013 class arrived between November 1 and 3, 2013 (class 4). We enrolled 105 (93%) of 113 class 4 recruits on November 25. The recruits were allowed to go on leave beginning January 20, 2014.

### Recruits

Recruits came from 28 of the 77 provinces of Thailand (including Bangkok) ranging in age between 20 and 31 years, with a median of 21 years (Table1). At least 37% of recruits had a high school or vocational school education, while 23% had only a primary school education (Table1). Smoking was very prevalent with 60% reporting being current smokers with 186 (70%) smoking half a pack per day or less. Only 2% had been vaccinated for influenza in the previous 12 months. Approximately 16% reported a medical condition at enrollment. The most common conditions were allergy (5%) and asthma (4%).

**Table 1:** Characteristics of enrolled Royal Thai Army recruits entering basic training by class

Variable	May 2012	November 2012	May 2013	November 2013	All classes
Total	*n* = 122 (%)	*n* = 113 (%)	*n* = 105 (%)	*n* = 105 (%)	*n* = 445 (%)
Age					
20	56 (45·9)	10 (8·8)	47 (44·8)	11 (10·7)	124 (28·0)
21	39 (32·0)	78 (69·0)	29 (27·6)	79 (76·7)	225 (50·8)
22–25	24 (19·7)	23 (20·4)	27 (25·7)	12 (11·7)	86 (19·4)
26–31	3 (2·5)	2 (1·8)	2 (1·9)	1 (1)	8 (1·8)
Education					
Elementary	29 (23·8)	16 (14·2)	30 (28·6)	27 (25·7)	102 (22·9)
Middle	41 (33·6)	52 (46)	43 (41)	40 (38·1)	176 (39·6)
High	20 (16·4)	18 (15·9)	10 (9·5)	18 (17·1)	66 (14·8)
Vocational	17 (13·9)	18 (15·9)	15 (14·3)	18 (17·1)	68 (15·3)
Bachelor	14 (11·5)	8 (7·1)	7 (6·7)	2 (1·9)	31 (7)
Other	1 (0·8)	1 (0·9)	0 (0)	0 (0)	2 (0·4)
Current smoker	68 (55·7)	77 (68·1)	55 (52·4)	67 (63·8)	267 (60)
Vaccinated for influenza in previous 12 months	3 (2·5)	2 (1·9)	0 (0)	4 (3·8)	9 (2·1)
Any medical condition*	21 (17·2)	12 (10·7)	14 (13·5)	23 (22·1)	70 (15·8)
Allergy	7 (5·7)	5 (4·5)	3 (2·9)	6 (5·8)	21 (4·8)
Asthma	4 (3·3)	1 (0·9)	6 (5·8)	5 (4·8)	16 (3·6)
Arthralgia	1 (0·8)	0 (0)	0 (0)	0 (0)	1 (0·2)
Diabetes	1 (0·8)	0 (0)	0 (0)	0 (0)	1 (0·2)
Epilepsy	1 (0·8)	0 (0)	1 (1)	0 (0)	2 (0·5)
Gastritis/ulcer	1 (0·8)	4 (3·6)	1 (1)	3 (2·9)	9 (2)
Cardio/hypertension	0 (0)	1 (0·9)	0 (0)	0 (0)	1 (0·2)
Hepatitis	2 (1·6)	1 (0·9)	1 (1)	2 (1·9)	6 (1·4)
Other conditions	3 (4·3)	0 (0)	1 (1·4)	6 (8·6)	10 (14·3)

* Some subjects report more than one medical condition. Therefore, the individual conditions do sum to the total with any medical condition.

### Single barracks/basic training (closed cohort)

Sixty-six URIs and 11 ILIs occurred in 56 class 1 recruits between weeks 2 and 10 of basic training (Table2). The rate was 6·9 per 100 recruit-weeks (95% CI: 5·5–8·6). The *P*-for-trend for the decrease in the weekly rate of illness over the course of basic training was 0·17. Fifty-five URIs among 48 class 2 recruits occurred between weeks 3 and 10 of basic training. The infection rate was 5·6 per 100 recruit-weeks (95% CI: 4·3–7·2). The *P*-for-trend for the decrease in the rate of illness during basic training was <0·0001. Twenty-eight URIs and 1 ILI among 29 class 3 recruits occurred between weeks 4 and 11 of basic training. The infection rate was 4·7 respiratory illnesses per 100 recruit-weeks (95% CI: 3·2–6·7). The *P*-for-trend for the decrease in the rate of illness during basic training was 0·01. Forty URIs and 3 ILIs among 41 class 4 recruits occurred between weeks 4 and 11 of basic training. The infection rate was 5·1 respiratory illnesses per 100 recruit-weeks (95% CI: 3·8–6·8). The *P*-for-trend for the decrease in the rate of illness during basic training was <0·01.

**Table 2:** Incidence of influenza-like illness or upper respiratory illness (URI) among Royal Thai Army recruits by week of basic training May 2012 to November 2013

	Class 1 (May 2012)			Class 2 (November 2012)			Class 3 (May 2013)			Class 4 (November 2013)		
	Training week	*n* = 122	pct	Week	*n* = 113	pct	Week	*n* = 105	pct	Week	*n* = 105	pct
1	6 May 2012	–	–	4 November 2012	–	–	5 May 2013	–	–	3 November 2013	–	–
2	13 May 2012	22	18	11 November 2012	–	–	12 May 2013	–	–	10 November 2013	–	–
3	20 May 2012	6	4·9	18 November 2012	16	14·2	19 May 2013	–	–	17 November 2013	–	–
4	27 May 2012	5	4·1	25 November 2012	10	8·8	26 May 2013	0	0	24 November 2013	11	10·5
5	3 June 2012	0	0	2 December 2012	0	0	2 June 2013	8	7·6	1 December 2013	0	0
6	10 June 2012	0	0	9 December 2012	0	0	9 June 2013	21	20	8 December 2013	9	8·6
7	17 June 2012	23	18·9	16 December 2012	29	25·7	16 June 2013	0	0	15 December 2013	0	0
8	24 June 2012	0	0	23 December 2012	0	0	23 June 2013	0	0	22 December 2013	23	21·9
9	1 July 2012	21	17·2	30 December 2012	0	0	30 June 2013	0	0	29 December 2013	0	0
10	8 July 2012	0	0	6 January 2013	0	0	7 July 2013	0	0	5 January 2014	0	0
11	15 July 2012	2*	1·6	13 January 2013	0	0	–	–	–	12 January 2014	0	0
12	–	–	–	–	–	–	–	–	–	19 January 2014	0	0
Total	–	77	–	–	55	–	–	29	–	–	43	–
*P*-for-trend 1**	Weeks 2–10		0·170	Weeks 3–10		<0·0001	Weeks 4–9		0·012	Weeks 4–11		0·006
*P*-for-trend 2***	Weeks 2–11		0·016	Weeks 3–11		<0·0001	Weeks 4–11		<0·001	Weeks 4–12		<0·001

pct, per cent.

* Two subjects had URI after subjects were permitted to go on leave and are not included in the rate for basic training.

** *P*-value for trend from Cohran–Mantel–Haenszel tests through last whole week prior to dismissal for leave after basic training.

*** *P*-value for trend from Cohran–Mantel–Haenszel tests through last week including date recruits went on leave after basic training.

### Post-basic training

The rate of respiratory illness among class 1 recruits upon their return to camp after basic training was 4·0 per 100 recruit-weeks (95% CI: 3·1–5·1). The rate ratio of respiratory illness during basic training relative to after basic training was 1·7 (95% CI: 1·29–2·29). The rate or respiratory illness among class 2 recruits upon their return after basic training was 2·3 (95% CI: 1·4–7·2). The rate ratio during basic training relative to after basic training was 2·46 (95% CI: 1·5–4·1).

### Laboratory-confirmed illness – influenza RT-PCR and multiplex PCR

**Figure 1:**
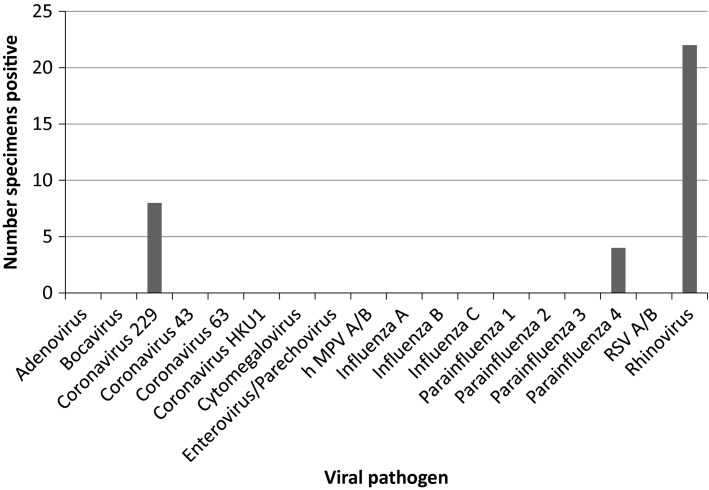
Three of 18 tested pathogens were detected in 31 of 76 respiratory specimens collected from recruits reporting a URI/ILI during the May 2012 Royal Thai Armay basic training. URI, upper respiratory illness; ILI, influenza-like illness.

Influenza RT-PCR was performed on all nasal/throat swab samples in the four classes. Only one sample (in class 3) was found to be positive for influenza virus (type B). Multiplex PCR was performed on 76 of the 77 nasal/throat swab specimens collected during basic training from class 1. Of 18 viral pathogens in the panel (including influenza A, B and C), three pathogens were detected among 31 (40·8%) of 76 specimens (Figure1). Rhinovirus was detected in 22 specimens, coronavirus 229 in eight specimens and parainfluenza 4 virus in four. Three recruits with rhinovirus had co-infections (two with coronavirus 229 and one with parainfluenza).

### Bed location

**Figure 2:**
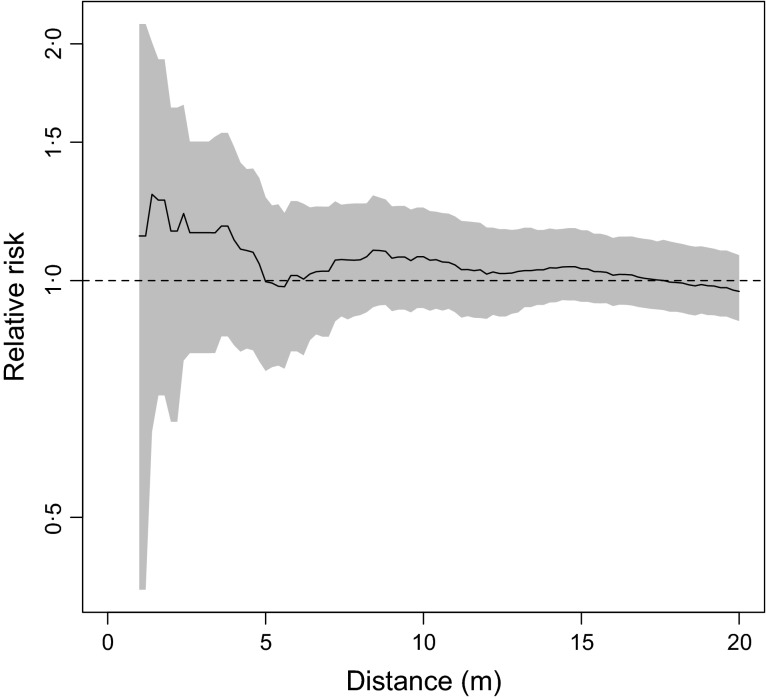
Relative risk of a URI/ILI case having a bed within a specified distance (in meters) of another case during the May 2012 Royal Thai Army basic training. URI, upper respiratory illness; ILI, influenza-like illness.

Figure2 shows the τ(*d*) clustering statistic over distances between 0 and 20 m between beds for class 1. We found some evidence of spatial dependence at very small spatial scales. Individuals were 1·2 times more likely to have a bed within 3 m of another case given they had a respiratory illness within a week of each other, relative to the probability that any two individuals had beds within 3 m of each other. However, the uncertainty in these estimates was wide (95% confidence intervals of 0·8–1·5). This value dropped to 1·0 at 5 m (95% confidence intervals of 0·8–1·3).

## Discussion

In this report on respiratory illness surveillance among new conscripts in a military barracks in the tropics, we found a high incidence of respiratory illness. Between 39% (class 4) and 46% (class 1) of recruits reported respiratory illness with incidence rates ranging from 4·7 to 6·9 per 100 recruit-weeks. The viral pathogens identified, rhinovirus, coronavirus, and parainfluenza virus, were compatible with URI symptoms prevalent in this study. At least one of these three pathogens was present in 40% of the samples collected. We found little febrile respiratory illness (i.e. ILI) and only one laboratory-confirmed influenza case even though influenza immunization was seldom reported among the recruits. Notably, at the time the first recruit class entered the barracks (May 2012), there was very little Influenza virus transmission.^18^ Thereafter, influenza circulated almost continuously throughout the study period in Thailand until December of 2013. However, at the time the last three recruit classes entered training, influenza transmission in Thailand was moderate (November 2012) to low (May and November 2013). Influenza activity in Thailand tends to peak after the beginning of the rainy season between July and September.^19^

Adenovirus, a pathogen that is recognized as the most common viral pathogen found in military barracks and responsible for a great proportion of respiratory illnesses among the U.S. military, was not identified among those specimens that underwent multiplex PCR.^20^ The paucity of influenza virus and adenovirus infections was most likely due to the fact that outbreaks of these pathogens were, simply by chance, not captured among the relatively small number of recruits in our study. With respect to influenza, the time the recruits came together in the camps did not happen to correspond to significant virus transmission in Thailand. A larger study involving thousands of individuals, particularly after the beginning of the rainy season, would be more likely to capture such outbreaks. However, it is also possible that the conditions within military barracks in the tropics may be less favorable for transmission of these viruses compared with more temperate climates, perhaps related to higher humidity and temperature. Larger studies among military recruits would need to be performed to clarify the burden of influenza and adenovirus among recruits in the tropics.

In this barracks setting during basic training, our results indicate the presence of infection dynamics typical of crowded conditions consistent with those seen in military barracks in the United States. In the U.S. military, the evidence suggests that most cases arise from internal transmission in which recruits infect one another rather than repeatedly being infected from outside the barracks.^21^ In one study in a U.S. barracks, the incidence of acute respiratory illness rose rapidly in the first few weeks, peaked in week 5, then declined by week 7 to below week 1 levels toward the end of training.^22^ The trend in our study of decreasing rates of infection over the course of basic training in three of the four classes supports the notion of initial seeding by new recruits infected from the outside with subsequent internal transmission leading to high initial rates followed by a decline due to depletion of susceptibles. Further support comes from the pattern of infections relative to bed location in the barracks. Though not significant, the likelihood of having a respiratory infection tended to increase with proximity to the bed of someone with URI/ILI. It should also be noted that recruits from each class were effectively sequestered from the outside during basic training. Though it is possible that the training instructors were allowed to come and go from the camp and, therefore, may have offered an opportunity for pathogens to be introduced from the outside, the instructors also slept in the barracks. Thus, it is likely that the infection dynamics reflected the seeding of infectious pathogens from recruits upon entering the class.

In the current study, soldiers in basic training were more likely to experience URI/ILI than soldiers who had already completed basic training in the same camps. This higher infection rate among recruits during basic training relative to more seasoned soldiers, those that have completed basic training, provides evidence that recruits in the RTA are particularly vulnerable to infectious respiratory diseases similar to what is seen in the U.S. military.^8^ The approximately twofold higher infection rates observed in RTA recruits in basic training is, however, at the lower end of the 2- to 16-fold higher rates seen in U.S. soldiers. The fact that these soldiers were conscripted may make our findings more generally applicable than had they gone through the self-selection that occurs among volunteer armies.

A few limitations of our study may have impacted the reliable inference regarding the infection dynamics in RTA training camps. For logistical reasons, we were unable to begin illness surveillance until 2–3 weeks had elapsed after initiation of basic training. Therefore, the first generations of infectious transmission were not observed to effectively characterize the possible initial increase in infection rates as may be expected under the conditions of internal transmission alone. We relied instead on the evidence provided by the tendency for decreasing transmission toward the end of training. The clustering statistic for distance between beds may be diluted due to other recruit activities done in close proximity such as eating, common toilets and instruction. Another limitation was that our study relied on syndromic surveillance. Thus, we had little opportunity to observe asymptomatic cases. Also, we could not be certain that pathogen exposure from the outside did not occur over the course of training. Furthermore, while a corpsman did routinely remind soldiers that they were to report any illnesses, some illnesses may have been missed. When illnesses were reported, they occurred in temporal clusters, suggesting that other, perhaps social factors, may have precipitated reporting. Finally, due to resource constraints, we were unable to perform multiplex PCR for all recruit classes.

While there are other settings with intense population mixing, such as schools, sports teams, prisons, and monasteries, the situation of new recruits in a military barracks represents a unique combination of sudden convergence of geographically diverse individuals, extended and continuous duration of mixing, and extreme physical exertion not seen in most other settings. The conditions for enhanced disease transmission in the RTA barracks may also have been different from barracks in more temperate climates such as the United States. For example, the RTA barracks were very well ventilated with wide open windows in the sleeping quarters. Also, other activities such as classroom training and eating occurred in the open air or under a roof only. It is possible that these factors could have potentially dampened intense internal transmission. Other simple interventions could also potentially be considered to mitigate transmission such as arranging sleeping cots foot to foot instead of head to head or organizing training activities and sleeping arrangements into discrete clusters.

Our findings are consistent with the dynamic of infections harbored by a few individuals upon entry into a military barracks and spreading over successive generations of transmission until susceptible individuals are depleted. Our study is effectively a random selection of an event that frequently takes place both in and out of the military. In this instance, the pathogens that caused infections produced mild disease. What is significant is that such congregations of people await the chance introduction of more virulent and/or communicable infections such as measles, mumps, norovirus, SARS-CoV, MERS-CoV, influenza, or emerging infections with significant potential for more debilitating disease or mortality.

## Author contributions

JWL and IKY contributed to study design, data analysis/interpretation, and manuscript writing. PB and HA contributed to study design, data acquisition, data analysis/interpretation, and manuscript writing. SS and KH contributed to data acquisition and manuscript writing. HS contributed to data analysis/interpretation and manuscript writing. RR, RGJ and RVG contributed to study design and manuscript writing. SF and CK contributed to data acquisition, data analysis/interpretation, and manuscript writing. The views expressed in this article are those of the authors and do not represent the official policy or position of the U.S. Department of the Army, Department of Defense, or U.S. Government.

## Supporting Information

**Appendix S1.** Description of cut-points used for defining weeks of basic training.
