# Supplementary material for: Elevated transmission of upper respiratory illness among new recruits in military barracks in Thailand
Source: Influenza Other Respir Viruses. 2015 Oct 13;9(6):308–14. doi: 10.1111/irv.12345 (PMC4605412; doi:10.1111/irv.12345)
Supplement: Supplementary file 1 — Appendix S1. Description of cut-points used for defining weeks of basic training. [file irv0009-0308-sd1.docx]

**Supplemental materials – cut points for weeks corresponding to basic training**

**Class 1:** the enrollment date 2012-May-14, corresponds to the week beginning the 2012-May-13 (week 2 of class 1 week sequence). The 17^th^ of July when the recruits went home was be the week beginning 2012-July-15 (week 11 of class 1 week sequence). The class 1 barracks period therefore was class 1 week sequence 2:11.

For the period corresponding to seasoned soldiers, the date the class returned from leave after basic was 2012-Jul-27 (week 12 of class 1 week sequence). Therefore the seasoned period began week 13).

**Class 2**: the enrollment date 2012-Nov-20, corresponds to the week beginning the 2012-Nov-18 (week 3 of class 2 week sequence). The 20^th^ of January, 2013 when the recruits went home was the week beginning 2013-Jan-20. Because the day the recruits went home is the beginning of the week, we did not include this week as the last week of basic. Instead, we chose the week before, the week beginning 2013-Jan13, corresponding to week 11 of the class 2 week sequence. Therefore, the class 2 barracks period was class 2 week sequence 3:11.

For the period corresponding to seasoned soldiers, the date the class returned from leave after basic was 2013-Jan-30 (week 13 of class 2 week sequence). Therefore the seasoned period began week 14 (the week beginning 2013-Feb-03). Note that there are 12 URI/ILI illnesses on the 30^th^ of January which is the date recruits came back from leave after basic training. We consider these to fall outside of both the barracks experience as well as the seasoned period.

**Class 3**, the enrollment date 2013-May-31, corresponds to the week beginning 2013-May-26 (week 4 of class 3 week sequence). The 11^th^ of July, 2013 when the recruits went home was the week beginning 2013-Jul-07 (week 10 of class 3 week sequence). The class 3 barracks period therefore was class 3 sequence 4:10.

**Class 4**, the enrollment date 2013-Nov-25, corresponds to the week beginning 2013-Nov-24 (week 4 of class 4 week sequence). The 20^th^ of January, 2012, when the recruits went home was the week beginning 2014-Jan-19 (week 12 of class 4 week sequence). The class 4 barracks period therefore was class 4 sequence 4:12.

**Supplementary materials: Estimator for *τ(d)***

We estimated *τ(d)* using the following estimator:

$$\pi_{1}\left( d \right)=\frac{\sum_{i=1}^{n_{c}} \sum_{j\neq i}^{n_{c}} {\boldsymbol{I}_{\boldsymbol{1}}(s}_{ij}<d, t_{ij}<7 days)}{\sum_{i=1}^{n_{c}} \sum_{j\neq i}^{n_{c}} \boldsymbol{I}_{\boldsymbol{2}}(t_{ij}<7 days)}$$

$$\pi_{2}\left( d \right)=\frac{\sum_{i=1}^{N} \sum_{j\neq i}^{N} {\boldsymbol{I}_{\boldsymbol{3}}(s}_{ij}<d)}{N(N-1)}$$

$$\hat{\tau}\left( d \right)=\frac{\pi_{1}\left( d \right)}{\pi_{2}\left( d \right)}$$

where $\pi_{1}\left( d \right)$ is calculated just using the individuals who had respiratory events and $\pi_{2}\left( d \right)$ is calculated using all individuals; *n_c_* is the number of respiratory events; *N* is the total number of individuals; *I_1_* is an indicator variable that is equal to one if individuals *i* and *j* had beds within distance *d* of each other and got sick within a week of each other and zero otherwise; *I_2_* is an indicator variable that is equal to one if individuals *i* and *j* got sick within a week of each other; *I_3_* is an indicator variable that is equal to one if individuals *i* and *j* had beds within distance *d* of each other.
